# Box-Counting Method of 2D Neuronal Image: Method Modification and Quantitative Analysis Demonstrated on Images from the Monkey and Human Brain

**DOI:** 10.1155/2017/8967902

**Published:** 2017-05-08

**Authors:** Nemanja Rajković, Bojana Krstonošić, Nebojša Milošević

**Affiliations:** ^1^Department of Biophysics, School of Medicine, University of Belgrade, Višegradska 26/2, 11000 Belgrade, Serbia; ^2^Department of Anatomy, School of Medicine, University of Novi Sad, Hajduk Veljkova 21, 21000 Novi Sad, Serbia

## Abstract

This study calls attention to the difference between traditional box-counting method and its modification. The appropriate scaling factor, influence on image size and resolution, and image rotation, as well as different image presentation, are showed on the sample of asymmetrical neurons from the monkey dentate nucleus. The standard BC method and its modification were evaluated on the sample of 2D neuronal images from the human neostriatum. In addition, three box dimensions (which estimate the space-filling property, the shape, complexity, and the irregularity of dendritic tree) were used to evaluate differences in the morphology of type III aspiny neurons between two parts of the neostriatum.

## 1. Introduction

The fractal analysis, a modern mathematical method of measuring complexity in nature [1], is initially derived from fractal geometry [2]. The concepts of fractal geometry are being used in diverse research areas [3] and particularly are proven to be useful tool as quantitative methods for image analysis in medical science [2, 4]. According to the fractal geometry, fractal analysis could be though, at present, as an experimental technique which calculates simple parameter: the fractal dimension (FD) of an object [5]. Previous work on fractal analysis represented traditionally a 2D analysis [5], focusing primarily on the object's border, structure, and indirectly object's function [2].

In fractal analysis, there are several different methods which calculate FD of object in a plane, but all of them can be summarized in two basic approaches: length-related and mass-related methods [3]. Using a different method has led to difficulties in comparing the results, because each method gives slightly different results when analyzing the same structure [2, 6]. As traditional caliper methods are typically time-consuming, the need for other practicable techniques emerges [5]. One of them is traditional box-counting method (BC method) which is based on concept of “covering” the image with rectangular coordinate grid [2]. Although this method is not suitable for measuring length, as well as other features of patterns, it is the best technique for estimating the FD. For that reason BC method is commonly used among other fractal techniques [2, 4, 5].

Now, traditional BC method is suitable method for measuring FDs of real objects [5, 7]. Thus, the first aim of this study was to modify standard BC method, using the appropriate scaling factor, image size, resolution, and rotation on 2D neuronal images from the monkey dentate nucleus. Having in mind modification procedure, this study could be seen as extension of previous investigations [5, 7], but here all conclusions were presented on asymmetrical neurons only. The second aim was to explore the differences in the morphology of the type III aspiny neurons from two parts of the human neostriatum, using standard and modified BC method.

## 2. Materials and Methods

Drawings of the Golgi-impregnated multipolar neurons of the monkey dentate nucleus were taken from the experimental data published in the book [8], in which is, also, described the histological procedure. The neuronal images were grouped according to Chan-Palay scheme of neuronal types [8]: 13 images of large “prickly” neurons, 18 images of boundary neurons, 24 images of asymmetrical neurons, and 22 images of “columnar” neurons [9].

Further, images from the neostriatum were collected from thirty human brains obtained from medicolegal forensic autopsies of adult bodies, free of neurological diseases [10]. The material was collected from 2008 to 2009 in the Center for Forensic Medicine, Toxicology and Molecular Genetics at the Clinical Center of Vojvodina (Serbia). As noted above, a total of 552 neurons (301 cells of the putamen and 251 cells of the caudate nucleus) were observed from both cerebral hemispheres [10]. From this population, 24 cells of the type III aspiny neurons [10] were selected for further analysis due to their unique dendritic arbor (low density of dendritic branching and giant dendritic domain [10]). The research was performed in accordance with the ethical standards defined by the 1964 Declaration of Helsinki, with the approval of the Ethics Committee of the University of Novi Sad (Faculty of Medicine, Serbia) [10].

### 2.1. Image Acquisition and Preprocessing

As we stated previously (Section 2), a total of 77 drawings from the monkey dentate nucleus were converted into digitized images using a scanner (Mustek 1200, Mustek System Inc., Taiwan) with a resolution of 600 dpi [9]. During the scanning, the size of each image was rescaled to A5 format due to the restriction of fractal software (particularly, BC method in* Image J*). To investigate BC methodology, we standardized this sample following similar number of primary dendrites, area, length, and density of dendrites.

**Figure 1:**
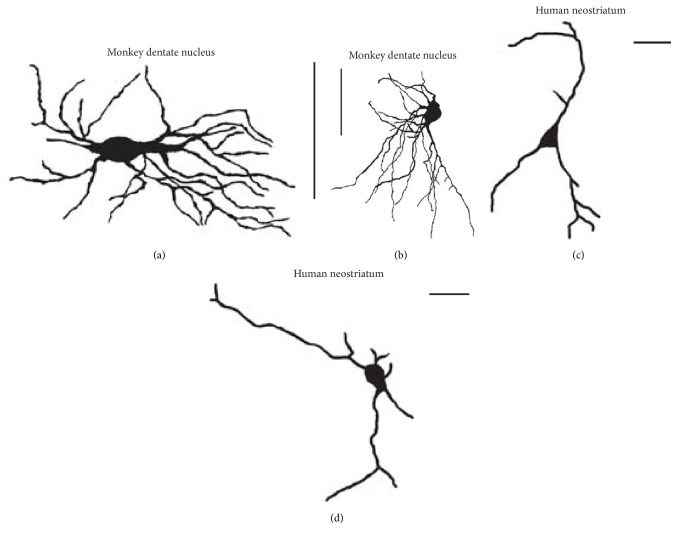
Representations of asymmetrical neurons from the monkey dentate nucleus: type 1 (a) and type 2 (b) cell. Original image can be found in Chan-Palay [8]. Representations of the type III aspiny neurons from the human neostriatum: image from the putamen (c) and caudate nucleus (d). All images are shown at 150 dpi with scale bar of 50 *μ*m.

Furthermore, taking into consideration width and height of the neuronal images, from the total of 77 images, 14 images were selected and classified in the way that the type 1 neurons, which included eight asymmetrical neurons, had a greater width than the height, and the type 2 neurons, which included six asymmetrical neurons, had a greater height than the width. Their schematic representation is shown in Figures 1(a) and 1(b).

In addition, 24 images of the type III aspiny neurons from the human neostriatum [10], 15 neuronal images from the putamen (Figure 1(c)), and 9 neuronal images from the caudate nucleus (Figure 1(d)), were analyzed with the standard and the modified BC method. After the histological sections of each obtained neuron were analyzed using a light microscope “Leica DC 100” (Leica Microsystems, Wetzlar, Germany) at a magnification of 40x, the images of neurons were transformed into digital images using the digital camera “Leica DC 100,” with the software package “Digital Camera Systems” (Leica Microsystems, Heerbrugg, Switzerland) [10, 11]. Depending on the somal size and dendritic arborization, each neuron was recorded in 4–20 focal planes. The digital images of these focal planes were loaded into* Image J* and using the “ZProject” command, the images were projected onto an image stack along the axis perpendicular to the image plane [10, 11].

Image processing was carried out using the public domain* Image J* software (https://imagej.nih.gov/ij/). By using the corresponding tools of that software, the axons and spines were removed from digital images of neurons, and each dendrite was filled with pixels.

### 2.2. Standard BC Method

*Segment-counting* method [5], a type of fractal analysis methods, is at the same time robust and time-consuming [7]. For this reason, the need for more handsome methods emerges, and* box-counting* [3] appears to be the method which suitably measures fractal dimensions of real objects [2, 3, 5, 12]. It is very similar (or sometimes, equal) to the idea from traditional calculus, when the “area of plane region” within any closed unformed boundaries should be measured [13], as the area is superimposed with a net of equivalent squares [14].

Traditional box-counting method “covers” the object with rectangular coordinate grid [3] and counts the number of boxes [2]. As each set of boxes is characterized by the square side* r*, the corresponding number of squares (*N*) necessary to cover the pattern is presented as a function of *r*. Fractal dimension (i.e., box dimension) is determined as the slope of the log-log relationship between *N* and *r*. Strictly mathematically “speaking,” the lower and upper box dimensions of a subset *F* ⊂ *R*^*n*^ are, respectively, defined by

(1) dim_BF=lim_δ→0log⁡NδF−log⁡δ,dim¯BF=limδ→0¯log⁡NδF−log⁡δ,

and if lower and upper values are equal, then the common value is referred to as the box-counting dimension of *F* and is denoted by

(2) $$\begin{array}{c} dim_{B}\left(\;F\;\right)=\underset{\delta \to 0}{lim}\frac{\log N_{\delta }\left(F\right)}{-\log\delta }, \end{array}$$

where *N*_*δ*_(*F*) can be the smallest number of cubes of side *δ* (naturally, in 3D) that covers *F* or the largest number of disjoint cubes of side *δ* with centers in *F* [15].

**Figure 2:**
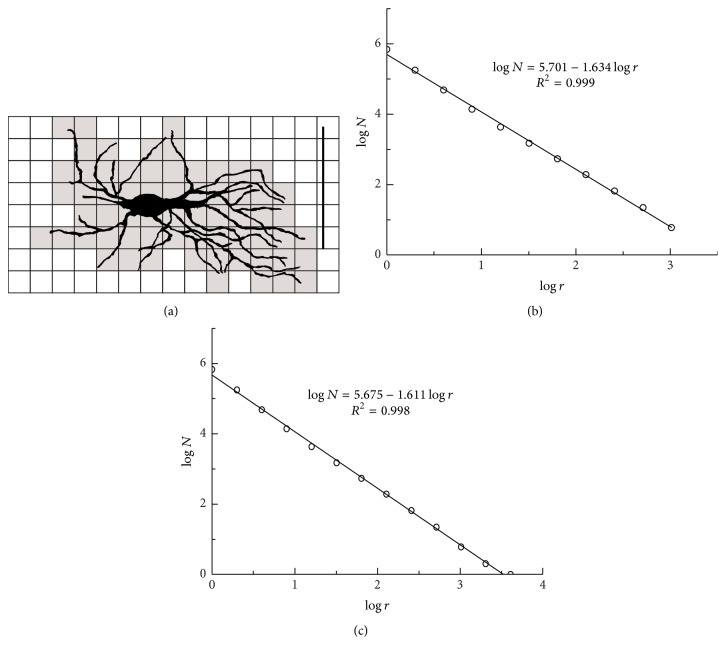
Application of the box-counting method to 2D image of type 1 neuron from the monkey dentate nucleus: (a) the whole image is covered with a set of squares and squares which cover dendrites are counted. (b) Log-log plot between numbers of squares (*N*) and square size (*r*) is fitted by a straight line. The square sizes (*r*) are taken by geometric progression. (c) Modification of square sizes (from 1 to 4096 pixels) illustrates interval of scaling and accurate BD. The linear equation between log⁡*N* and log⁡*r* is shown in upper part of graphs, where *D*_*B*_ is the absolute value of the slope and *R* is the corresponding correlation coefficient.

When BC method is applied on digitized images, it covers the image with a grid of square cells (with cell size *r*), where the cell size is expressed as the number of pixels (Figure 2(a)). The number of squares *N*(*r*) needed to cover the image is given by a power law

(3) $$\begin{array}{c} N\left(\;r\;\right)=const\cdot r^{-D_{B}}, \end{array}$$

where *D*_*B*_ is the box dimension (BD in further text), obtained as an absolute value of the slope of the log-log relationship between *N*(*r*) and *r* [3] (Figure 2(b)).

### 2.3. The Image of the Neuron and BC Method

#### 2.3.1. Modification of Box Sizes

Even though mathematical fractal requires infinite orders of magnitude of the scaling [1], various structures in nature have a finite number of decades between a high and a low cut-off scale [2]. For instance, previous studies [5, 16] promote a hypothesis that the 2D neuronal images could be considered fractal over several decades of scale, if the box sizes are scaled as a power of 2. In addition, they present results of standard BC method, using arithmetical, geometrical, and random progression of box sizes [5, 16] where statistical evaluation of the correlation coefficient of fitted line has shown that it is different from zero with a very high significance (*p* < 0.0001). Despite very high value of correlation coefficient, choosing the size of boxes as a finite increasing geometric progression, compared to arithmetical progression, represents better solution of fitting problem, because in this case the starting object will fulfill all conditions of fractal analysis [5].

Finally, standard BC method should be modified as follows: the box sizes should be taken from 2^0^ to 2^*k*^ pixel, where *k* is the value for which *N* is equal to one (Figure 2(c)). In that case, besides different value of *D*_*B*_, the relationship between log⁡*N* and log⁡*r* was linear on more than two decades of the range [2], when correlation coefficient of fitted line was statistically evaluated.

#### 2.3.2. Influence of the Neuronal Image Size and Resolution

The fractal analysis of the same cells at different resolutions returned different FD values, even when all other parameters are kept constant [2]. What is more, in their previous work Jelinek et al. [17] have concluded that cells scanned at low resolution had higher values of FD than those obtained at high resolution. Previous study tested this hypothesis in one manner [7]: 14 images of asymmetrical neurons from the monkey dentate nucleus were printed on A4 paper (the size of each image was 13 × 18 cm) and image was scanned from resolution of 100 dpi to 1100 dpi. The choice of final resolution was restricted by type of a scanner (Mustek 1200, Mustek Systems Inc., Taiwan). Results of this study suggested that mean *D*_*B*_ increased with resolution, with extremely low value of the slope; therefore* the resolution does not influence BD value of the image* [7].

Present study shows another step in investigating influence of image resolution (and, thus, different image size) on value of *D*_*B*_. The same sample of images were initially scanned at 600 dpi, and for each image, resolution was digitally changed (increased or decreased). Thus, we gained two samples of asymmetrical neurons (type 1 and type 2) with the same interval of resolution as previous study (100 dpi–1100 dpi). Results confirmed conclusions presented in [7]: there was an increase in *D*_*B*_ with the resolution, but again, with very low value of the slope (10^−4^ for type 1 and 0.9 × 10^−4^ for type 2). For both types of asymmetrical neurons results suggested that high resolution would maximize the resemblance between the digital and original drawings [7].

#### 2.3.3. Image Rotation

The picture exhibits* rotational symmetry *if rotation by a specific angle around some central axis point can return the picture to its original configuration [18], and it is reasonable to imagine that the* D*_*B*_s of such picture in these positions have the same value. As for neuronal image, some findings reported unexpected results for images of neurons which possess lack in strong radial symmetry, that is,* nonstellate neurons* [18] or, that is,* asymmetrical neurons*. Previous study proposed possible explanation on how to perform exact calculation of *D*_*B*_ [7]: first, the axis of rotation was created connecting two distant points of dendritic field area around the neuron [19]. Then, all images were continuously rotated from 0° to 360°, increasing the angle by 15°. *D*_*B*_ either increases or decreases and maximal (or minimal) *D*_*B*_ was noticed in 45°, 135°, 225°, and 315°. The final *D*_*B*_ was calculated as the mean of these four values.

**Figure 3:**
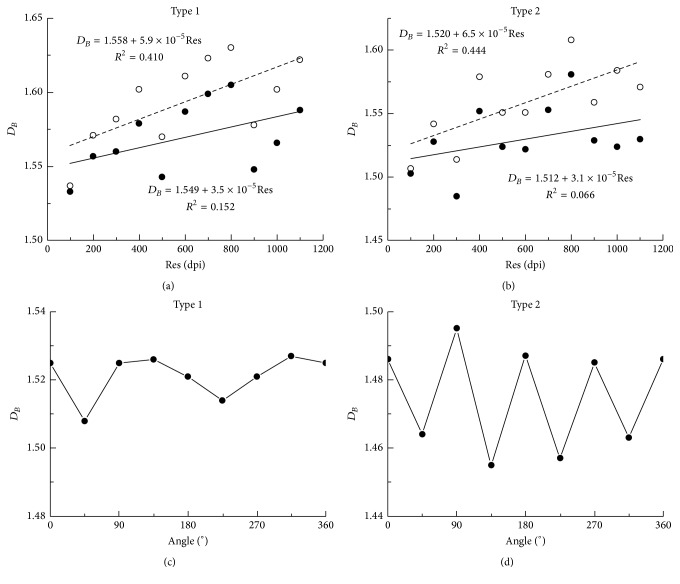
(a, b) Plots of (*D*_*B*_)_bin_ versus resolution for type 1 (a) and type 2 (b) neurons shown in Figure 1: full line is for scanned images and dashed line is for digital changes. The equations between *D*_*B*_ and resolution, as well as correlation coefficients are inscribed in each graph. (c, d) Changes in (*D*_*B*_)_bin_ with an angle of rotation (*α*) for type 1 (c) and type 2 (d) asymmetrical neurons of monkey dentate nucleus.

Thus, to calculate accurate *D*_*B*_ for asymmetrical neuronal images, we propose another modification of the BC method:* each image should be analyzed for symmetry*. The axis of rotation should be constructed and the image should be rotated by four angles (45° +* kπ*/2, where* k* = 0, 1, 2, and 3). In each position, apparent *D*_*B*_ should be recorded and final (or precise) *D*_*B*_ will be the mean of these values. Figures 3(c) and 3(d) illustrate this procedure for type 1 and type 2 asymmetrical neurons from the monkey dentate nucleus.

#### 2.3.4. Image Types and Corresponding BDs

When 2D RGB or grayscale image of the neuron is quantified by BC method, two characteristic image presentations are recognized:* binary* and* outline* (previously known as “silhouette” [3]) image. In any software for image analysis, the binary image represents compression of an initial image to two values (i.e., black and white values). Consequently, the outline image was created when one-pixel wide outline of foreground objects in a binary image was generated.

Previous study provides explanation on how *D*_*B*_ can estimate an object's projection in plane [5]. Theoretically, when size of squares was reduced by two, the number of boxes multiplies previous number by 4, and *D*_*B*_ was equal to 2 with coefficient of determination equal to one. Thus, for 2D neuronal image, the value of *D*_*B*_ estimates the area of its projection [5], but precisely it can be stated that *D*_*B*_ estimates* space-filling* property of the neuron. Exactly, *D*_*B*_ estimates how neuronal projection fills the plane defined by image size. Then again, the border of binary image (i.e., outline image), evaluates the irregularity in the shape of the image or precisely the value of *D*_*B*_ shows how this value deviates from values of classic geometric figures or more complex forms.

There is another type of image, commonly used in earlier phase of digital image analysis, when images of neurons are drawn by camera lucida throughout dendritic axis [14]. It is known as* skeleton* image of the neuron [14], since they represent only the dendritic branching and do not reflect the other characteristic of complexity or border roughening [2, 20]. In most software, command* skeletonize* works only with binary 2D images, removing pixels from the edges of objects until they are reduced to single-pixel-wide shapes.

**Figure 4:**
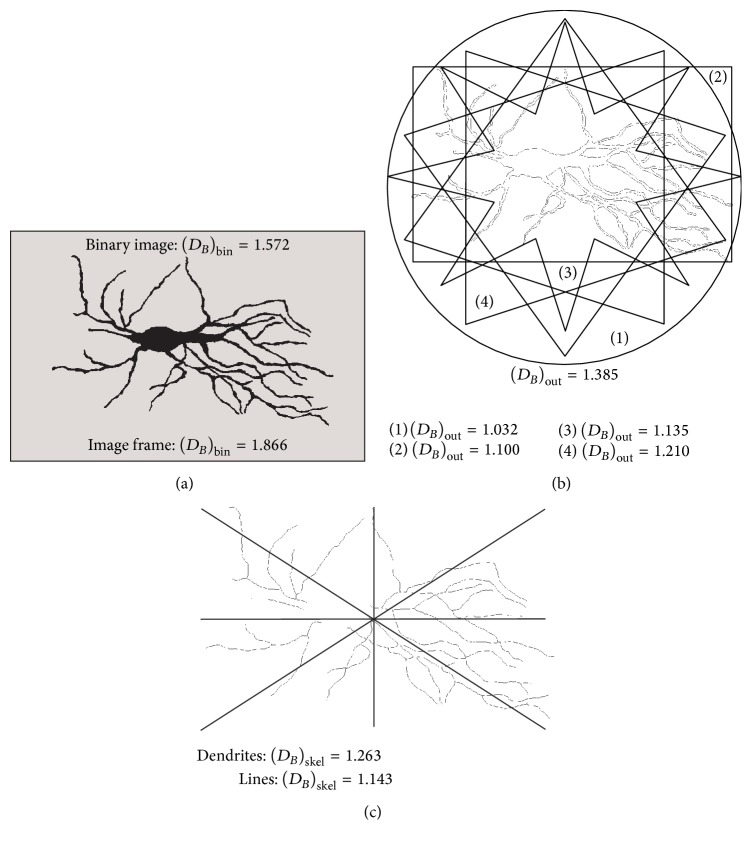
The binary (a), outline (b), and skeleton (c) images of asymmetrical neuron shown in Figure 1(a). For each image corresponding BD is presented along with theoretical values from which calculated BD has been compared, in order to evaluate three morphological properties of the neuron.

Traditional BC method was modified when 2D neuronal images have been analyzed by using skeleton process [5], particularly when dendritic branching of neurons with thick dendrites and large cell bodies have been investigated [18]. The cell body on the binary image of neuron was digitally removed and remaining dendrites were sublimated in single pixel line while remaining artifacts were deleted [2, 5, 14]. The analysis of such image or *D*_*B*_ of this kind of neuronal image precisely estimates both, dendritic branching pattern and dendritic aberration [5].  Figure 4 illustrates three BDs, which evaluate the space-filling property, shape, dendritic aberration, and complexity of dendritic tree for type 1 asymmetrical neurons from the monkey dentate nucleus.

## 3. Results

The morphology of 24 images from the human neostriatum (i.e., type III neurons) was analyzed by BC method in order to explore (i) the difference in BDs obtained by standard and modified BC method and (ii) the possible difference in BDs between two parts of the human neostriatum (putamen and caudate nucleus). BC method was done using* Image J*, after each image was saved in three different formats: binary (Process: Binary → Make binary), outline (Process: Binary → Outline), and skeleton (Process: Binary → Skeletonize) image and corresponding BDs were calculated. The skeleton process in* Image J* uses a thinning algorithm, thoroughly explained in the study of Zhang and Suen [21].

Analysis of the calculated *D*_*B*_s depends on whether the distribution is normal or not [22]. Regardless of the fact that the type III neurons were selected from normally distributed population [10], the number of neurons was relatively small and character of the distribution could be tested with two statistical parameters: skewness (*a*_3_) and excess of distribution (*e*) [5]. In brief, the intervals of distributions are estimated when *a*_3_ and *e* were divided by the corresponding mean square errors (*σ*_3_ and *σ*_4_). If the absolute value of the quotients *σ*_3_/*a*_3_ and *σ*_4_/*e* is less than or equal to 2, then the data distribution can be considered as normal [5].

Table 1 shows values of *a*_3_ and *e* for three different *D*_*B*_s calculated with standard and modified box-count method. As the absolute ratios (*σ*_3_/*a*_3_ and *σ*_4_/*e*) are smaller than the critical value of 2 in all cases, thus, the calculated *D*_*B*_s can be expressed by the mean values and standard errors. As can be seen, the mean (*D*_*B*_)_bin_ and (*D*_*B*_)_skel_ were higher by standard BC method than the modified, while opposite conclusion can be drawn for mean (*D*_*B*_)_out_. Moreover, the difference between two methods was 0.4% for (*D*_*B*_)_skel_, 0.7% for (*D*_*B*_)_out_, and 2.7% for (*D*_*B*_)_bin_. Further, only for (*D*_*B*_)_bin_ the difference was statistically significant.

The second task of this study was done by calculating three BDs using modified BC method.  Table 2 shows means and standard errors for (*D*_*B*_)_bin_, (*D*_*B*_)_out_, and (*D*_*B*_)_skel_ for the neuronal images of the putamen and the caudate nucleus. It looks that the images of neurons in the caudate nucleus have larger value of mean (*D*_*B*_)_bin_ than the images in the putamen, but for all three DBs no significant differences were found (Table 2).

## 4. Discussion

The extension of the concepts of fractal geometry toward the biomedical sciences has led to significant progress in understanding complex functional properties and structural features [23–29]. Once fractal geometry was formulated, many neuroscientists adopted fractal analysis as an appropriate method for objective quantitative analysis of neuronal structures [2, 30]. One of the advantages of using fractal analysis is its capacity to make a difference among neurons that differ in the complexity of their dendritic and axonal branching patterns [2, 3, 7].

### 4.1. Modified BC Method

As Section 2.2 explains mathematical background of standard BC method, this paper presents its modification, particularly when 2D image of neuronal projection has been quantified (Section 2.3). Standard BC method was improved with the appropriate scaling factor (Section 2.3.1) and the image size and resolution have been explored (Section 2.3.2). Along with previous, this paper improves conclusion presented in the study of Ristanović and coauthors [18], regarding image rotation and correct *D*_*B*_ calculation (Section 2.3.3). The influence of image size, resolution, and rotation has been presented on images of asymmetrical cells from the monkey dentate nucleus.

The Methods section in this text shows significance of *D*_*B*_ calculated for different presentations of the same neuronal image. Precisely, *D*_*B*_ evaluates the space-filling property, perimeter of the neuron shape, and irregularity of dendrites.

In previous study [31], images from several mammalian spinal cords were investigated, with proposed scaling factor, and results undoubtedly showed that images from rat's and cat's spinal cords should be investigated, mainly, as binary images [31]. Another study summed up investigation of 76 images from the adult human dentate nucleus [5] with three types of BDs. (*D*_*B*_)_bin_ was subjected to the size of the dendritic field (*A*_DF_) and (*D*_*B*_)_out_ was subjected to the circularity ratio (*M*). The results demonstrated the fact that BDs are more sensitive than the size of dendritic field and circularity ratio [5].

### 4.2. Aspiny Neurons of the Human Neostriatum

To illustrate proposed modification of the BC method, we choose images of aspiny neurons from the human neostriatum, precisely images of the type III neurons. Our pool of cells represents statistically small sample (less than 30), but these could be expected as previous study [10] postulates that this type of cells consists up to 10% of the whole population. However, this sample was enough to demonstrate the difference between standard and modified BC method. The difference was obvious (i.e., statistically significant) when binary images have been compared (Table 1).

Another task of this study was to investigate morphological differences of aspiny neurons between two parts of the neostriatum. Previous study [10] reported differences between neurons of the putamen and the caudate nucleus when dendritic field area and density of the dendritic trees were analyzed. This paper analyzes space-filling property, shape of the neuron, complexity, and irregularity of dendrites with modified BC method. Results look unequivocal: the binary BD is higher for the neuronal images of the caudate nucleus (while outline and skeleton BDs are lower) than the images of the putamen (Table 2), but without statistical significance. Such information leads us to the conclusion: either this sample of neurons was too small or the type III aspiny neurons from two parts of human neostriatum have similar morphology (i.e., the space-filling property and shape of the neuron, as well as dendritic complexity and aberration). However, we believe that further study with the large number of the type III neurons would provide more precise conclusions.

## 5. Conclusion

The main aim of the fractal analysis is to calculate the FD of an object and to ascertain the significance of the obtained value in terms of the complexity of the object. As for 2D image of the neuron, FD should quantify its morphology on the basis of how image is presented. However, the present study shows importance of image preprocessing, particularly when FD is calculated by BC method. BC modifications could be very important having in mind that BC method is, today, the most common procedure for calculation of FD.

## Figures and Tables

**Table 1:** Box-counting analysis of 24 images of the type III aspiny neurons from the adult human neostriatum. The values of skewness (*a*_3_) and excess (*e*) for binary ((*D*_*B*_)_bin_), outline ((*D*_*B*_)_out_), and skeleton ((*D*_*B*_)_skel_) box dimensions, calculated by standard and modified BC method. *t*_*c*_ is calculated *t*-value and *p* is the significance level.

Box-count dimensions		*a*_3_^*∗*^	*e* ^*∗*^	Mean ± SE	*t*_*c*_^*∗*^	*p*
(*D*_*B*_)_bin_	Standard	−0.292	−1.352	1.43 ± 0.01	4.074	<0.05
	Modified	0.401	−1.930	1.393 ± 0.008		

(*D*_*B*_)_out_	Standard	−0.335	−1.094	1.186 ± 0.009	0.740	>0.05
	Modified	0.550	−0.382	1.194 ± 0.007		

(*D*_*B*_)_skel_	Standard	0.369	−0.821	1.086 ± 0.008	0.359	
	Modified	0.452	−0.992	1.082 ± 0.008		

^*∗*^ *σ*_3_ = 0.452, *σ*_4_ = 0.768, *t*_0.05_ = 2.069, *t*_0.01_ = 2.807, and *t*_0.001_ = 3.767.

**Table 2:** Three box dimensions ((*D*_*B*_)_bin_, (*D*_*B*_)_out_, and (*D*_*B*_)_skel_) of the type III neuronal images from two parts of the human neostriatum. *t*_*c*_ is calculated *t*-value and *t*_*t*_ is tabulated *t*-value on *p* = 0.05.

Box dimension	Type III neurons		*t*_*c*_	*t*_*t*_
	Caudate nucleus	Nucleus putamen		
(*D*_*B*_)_bin_	1.40 ± 0.02	1.387 ± 0.008	1.092	2.074
(*D*_*B*_)_out_	1.19 ± 0.01	1.20 ± 0.01	0.379	
(*D*_*B*_)_skel_	1.07 ± 0.01	1.09 ± 0.01	0.395	
